# Assessment of the Endophytic Fungal Composition of *Lactobacillus plantarum* and *Enterococcus faecalis*-Fermented *Astragalus membranaceus* Using Single-Molecule, Real-Time Sequencing Technology

**DOI:** 10.3389/fvets.2022.880152

**Published:** 2022-04-28

**Authors:** Liheng Zhang, Xianghui Li, Xinghui Song, Chuanzhou Bian, Xiangtao Kang, Junqiang Zhao, Hongxing Qiao, Yanzhang Gong

**Affiliations:** ^1^Key Laboratory of Agricultural Animal Genetics, Breeding and Reproduction of Ministry of Education, College of Animal Science and Technology, Huazhong Agricultural University, Wuhan, China; ^2^College of Veterinary Medicine, Henan University of Animal Husbandry and Economy, Zhengzhou, China; ^3^College of Animal Science and Technology HAU, Henan Agricultural University, Zhengzhou, China; ^4^Henan Tianhao Hongfa Biotechnology Co., Ltd., Zhengzhou, China

**Keywords:** *Astragalus membranaceus*, fermentation, endophytic fungi, SMRT sequencing, 18S rRNA

## Abstract

Endophytic fungus represents microorganisms existing within the healthy plant organs, which can significantly influence metabolic product production in plants, a process with great research value and broad prospects for development. To investigate the effect of fermentation with probiotic cultures on the endophytic fungal diversity and composition of *Astragalus membranaceus*, we used single-molecular, real-time sequencing (Pacific Biosciences) for 18S ribosomal RNA (rRNA) sequencing. The results showed that the endophytic fungi of *A. membranaceus* mainly belonged to *Aspergillus, Penicillium, Cystofilobasidium, Candida, Guehomyces*, and *Wallemia*. Furthermore, the endophytic fungal diversity and abundance of *A. membranaceus* were more variable after fermentation with *Enterococcus faecium and/or Lactobacillus plantarum*. Our data lays a solid and comprehensive foundation for further exploration of endophytic fungi from *A. membranaceus* as potential sources of functional compounds.

## Introduction

*Astragalus membranaceus* (Fisch.)Bge. as part of one of the largest genera in the *Leguminosae* family, has been used as a traditional tonic for promoting digestion and metabolism, enhancing immunity, and accelerating injury/wound healing (1, 2).

Astragalus is a traditional Chinese herbal medicine that has been widely used by humans for hundreds of years. There are few studies on the use of *A. membranaceus* in livestock. With the rapid development of intensive livestock production, there is an urgent need for sustainable and environmentally friendly practices in this area. *A. membranaceus* is also utilized to be the supplementary agent in anti-tumor treatment, which exhibits diverse effects like anti-bacterium, anti-virus, antioxidation, anti-inflammation, and hidroschesis. However, the extraction of *A. membranaceus'* functional ingredients is limited because of plant cell wall recalcitrance, while microbial fermentation offers a possibility to improve *A. membranaceus* utilization efficiency. Previous studies show that *A. membranaceus* fermentation by *Lactobacillus plantarum* (*L. plantarum*) and/or *Enterococcus faecium* (*E. faecium*) can increase *Astragalus* polysaccharides, flavonoids, and saponins contents, whereas *A. membranaceus* after fermentation has high microbial abundance and diversity (3). It can be speculated that the fermentation of *A. membranaceus* elevates the content of active components by affecting its endophytic fungi.

Microorganisms exist in almost all living and non-living niches on the earth, like in thermal vents, deep rocks, sediments, or under extreme conditions like marine habitats and deserts (4). Endophytes are microorganisms (mostly bacteria and fungi) that reside in plants during one part or the entire life cycle with no instigation of distinct infection symptoms or visible manifestation of diseases in their hosts (5, 6). Endophytic fungi are meiosporic or mitosporic ascomycetes and present great biological diversity, and at least one species exists in every plant (7). In addition, microorganisms also produce metabolites to promote development, repellents for pests and insects, protectors, and antibacterial agents to resist plant pathogens under conditions like stress (8, 9). Moreover, investigations have clearly demonstrated that plant survival and health are strongly dependent on their endophytes (10, 11). Endophytic fungi may generate specific secondary metabolites, and these may be utilized in fields like pharmaceutics and agriculture (8). Paclitaxel (Taxol), a commonly used antitumor agent, can be generated by *Taxus brevifolia* and subsequently *via Taxomyces andreanae* (also an endophytic fungus), which accounts for a distinct example for the production of endophyte-plant metabolites (4).

Recently, research mainly aimed to isolate endophytic fungi from *A. membranaceus* in China and examine their antimicrobial activities. Several reports have stated that *A. membranaceus* endophytic fungal species can generate diverse secondary metabolites with bioactivity (12). However, endophytic fungal species in fermented *A. membranaceus* have never been studied. Here, we aimed to detect and compare endophytic fungi in *A. membranaceus* under fermentation *via L. plantarum* and *E. faecium* by' single-molecular, real-time (SMRT) sequencing (Pacific Biosciences), the third next-generation sequencing platform.

## Materials and Methods

### Fermented *Astragalus* Synthesis

*Astragalus membranaceus* dried roots were provided by Gansu Huisen Pharmaceutical Co., Ltd. (Minxian County, Gansu Province), whereas the isolation and preservation of *E. faecium* (CGMCC 1.130) and *L. plantarum* (CGMCC 1.557) were completed at the China General Microbial Species Preservation Center (CGMCC, Beijing, China). The preparation of fermented *A. membranaceus* was as previously reported (3). In short, *A. membranaceus* was first ground into powder and passed through the 100-mesh filter. Thereafter, we classified 10,000 g dried *A. membranaceus* powders into 4 groups: A, B, C, and D. This work inoculated group A by 10^6^ colony-forming units (CFU)/g *E. faecium*, group B by 10^6^ CFU/g *L. plantarum*, group C with 10^6^ CFU/g *E. faecium* combined with *L. plantarum*, and group D by lactobacillus selector (agar) medium as a blank control. Each group had three replicates. Then, fermentation was carried out in plastic bags (35 × 45 mm, Zhejiang Jinhu Company, China), followed by vacuum pumping and sealing by vacuum packaging of the bags. Thereafter, the mixture was fermented under 37°C and anaerobic situation for a 6-day period for producing the fermented *A. membranaceus*. On the 6th day of fermentation, the four groups were sampled. Three repetitive samples were collected in each group and mixed into one sample for sequencing. This study labeled the samples as A, B, C, and D.

### DNA Extraction

Four samples were collected from each group (200 mg each) after fermentation, followed by immediate freezing under −196°C to extract the DNA subsequently. Afterward, this study utilized the Qubit 2.0 fluorometer (Invitrogen, Carlsbad, CA, USA) for quantifying the DNA samples. We evaluated the quality of the extracted DNA through 0.8% agarose gel as well as 260/280 mm spectrophotometry. Before further analysis, the DNA extracted was preserved under −20°C. PCR was performed to amplify 18S ribosomal RNA (rRNA) of fungi, followed by being sequenced by SMRT barcodes using the primers below, 27F (5′-CCGTGTTTCAAGACGG-3′, forward) and 1492R (5′-CTTGGTCATATA GAGTAA-3′, reverse). The 25-μl amplification volume was prepared, containing 5 μl of 5 × GC buffer, 5 μl of 5 × reaction buffer, 2 μl of 2.5 mm dNTP, 2 μl DNA template, 1 μl of 10 μm reverse primer, 1 μl of 10 μm forward primer, 0.25 μl Q5 DNA polymerase, and 8.75 μLddH_2_O. The PCR was implemented as follows: 2-min predenaturation step under 98°C; followed by 15-s under 98°C, 10-s under 55°C and 30-s under 72°C for 30 cycles; and eventual 5-min elongation under 72°C. The whole 18S rRNA length was sequenced by the Shanghai Passenger Company.

### Data Analysis

The original reads generated by cyclic consensus sequencing were used to render the accuracy of prediction up to 90%. We extracted high-quality sequences using the QIIME (Shanghai Personalbio Technology Co., Ltd, Shanghai, China) package (Quantitative Insights into Microbial Ecology, v1.8, https://docs.qiime2.org/2019.4/tutorials/) (13). In addition, we utilized USEARCH (Shanghai Personalbio Technology Co., Ltd, Shanghai, China) (v5.2.236, http://www.drive5.com/usearch/) for excluding chimeras and clustering the clean sequence data into operational taxonomic units (OTUs) at the similarity degree of 97% (14). Later, this work adopted UNITE fungal ITS database (https://unite.ut.ee/) for sequence comparison through blast search (15).

Moreover, ACE, Chao 1, Simpson, and Shannon indicators were utilized to estimate the alpha-diversity. The R software (Bell Lab, Lucent Technologies, Beijing, China) was also employed to classify data from 50 species with the highest abundances, later, the heat map was generated. This study also carried out principal component analysis (PCA) with R software (16).

The original sequence readings were imported into the National Center for Biotechnology Information Sequence Read Archive with accession No. SAMN07411593-SAMN07411601.

## Results

### Diversity of Endophytic Fungal Species Originating From Fermented *A. membranaceus*

Full-length 18S rRNA gene was subject to SMRT sequencing for obtaining the precise fungal profiles from all four samples. We acquired altogether 99,923 sequence readings in the four samples, resulting in 24,980 readings per sample on average. Thereafter, this study determined Chao 1, ACE, Simpson, and Shannon indicators, together with the diversity of fungal species in each group is shown in Table 1. According to Table 1, Chao1, ACE, and Shannon indicators of group B were the highest, while the Simpson index showed little difference among the four groups.

**Table 1:** Estimated alpha-diversity indices of 18S ribosomal RNA (rRNA) gene libraries for four samples used for sequencing.

**Sample**	**ACE**	**Chao 1**	**Shannon**	**Simpson**
A	229.00	229.00	4.73	0.92
B	303.00	303.00	5.18	0.93
C	227.00	227.00	5.09	0.94
D	243.00	243.00	4.80	0.91

*Astragalus membranaceus samples under fermentation with Enterococcus faecium (A), Lactobacillus plantarum (B), E. faecium + L. plantarum (C), and LBS (D) on day 6*.

### Comparative Analysis

**Figure 1:**
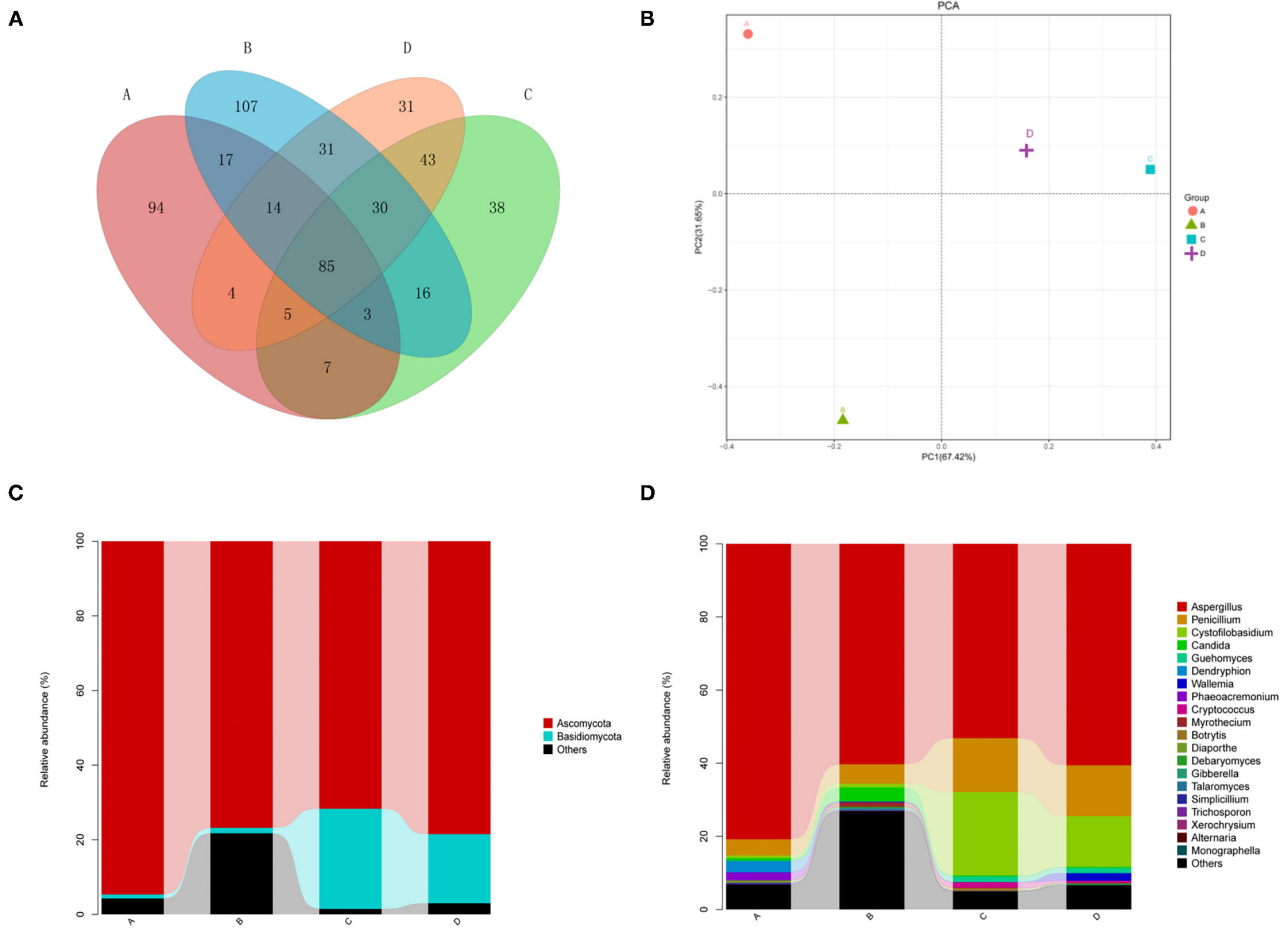
**(A)** Venn diagram showing the distributions of operational taxonomic units (OTUs) across 4 groups. Astragalus membranaceus samples under fermentation with Enterococcus faecium. E. faecium (A), Lactobacillus plantarum (B), E. faecium + L. plantarum (C), and LBS (D) on day 6. **(B)** Two-dimensional principal component analysis plot of the samples. *A. membranaceus* samples under fermentation with *E. faecium* (A), *L. plantarum* (B), *E. faecium* + *L. plantarum* (C), and LBS (D) on day 6. **(C)** Phylum levels in samples. *A. membranaceus* samples under fermentation with *E. faecium* (A), *L. plantarum* (B), *E. faecium* + *L. plantarum* (C), and LBS (D) on day 6. **(D)** Genus levels in samples. *membranaceus* samples under fermentation with *E. faecium* (A), *L. plantarum* (B), *E. faecium* + *L. plantarum* (C), and LBS (D) on day 6.

The comparative analysis of the total OTUs of groups A, B, C, and D was carried out (Figure 1). Altogether, 1,002 OTU types were detected from all groups, and 85 OTU types were common across them. Eighty-eight OTUs were common among groups A, B, and C, whereas 107, 160, and 163 OTU types of group D were specifically associated with groups A, B, and C, respectively.

### Principal Component Analysis (PCA)

Two-dimensional PCA at the genus level was carried out for evaluating different fungal community structures among 4 groups (Figure 1). PC1/PC2 of the PCA at the genus level explained 67.42 and 31.65% of the total variation, separately. As revealed by PCA, all four groups were grouped separated, and groups C and D were closer than the others, indicating that the fungal communities of groups C and D were similar.

### Phylum-Level Composition of the Endophytic Fungal Community

Our results showed that the dominant fungal phyla of *A. membranaceus* were *Ascomycota* and *Basidiomycota* (Figure 1). The abundances of *Ascomycota* in 4 groups were 94.7, 76.8, 71.7, and 78.5%. The abundances of *Basidiomycota* in the 4 groups were 1.1, 1.5, 26.9, and 18.5%. The abundances of unclassified phyla in the 4 groups were 4.2, 21.7, 1.4, and 2.9%. These results indicated that the fermentation of *A. membranaceus* by *L. plantarum* or *E. faecium* had a greater impact on endophytic fungi of *A. membranaceus*, with a significantly decreased abundance of *Basidiomycota*. Furthermore, *L. plantarum*-fermented *A. membranaceus* significantly increased the abundance of unclassified phyla.

### Genus Level Compositions of the Endophytic Fungal Communities

According to Figure 1, the dominant fungal genera of *A. membranaceus* were *Aspergillus, Penicillium, Cystofilobasidium, Candida, Guehomyces*, and *Wallemia*. The abundances of *Aspergillus* in 4 groups were 80.8, 60.3, 53.1, and 60.6%. The abundances of *Penicillium* in the 4 groups were 4.4, 5.3, 14.7, and 13.9%. The abundances of *Cystofilobasidium* were.7, 1.0, 22.8, and 13.8%. The abundances of *Candida* were 0.7, 3.8, 0.3, and 0.3%. The abundances of *Guehomyces* were 0.1, 0.1, 1.5, and 1.4%. The abundances of *Wallemia* were 0.0, 0.2, 0.1, and 2.1%. The abundances of unclassified genera were 4.9, 26.1, 2.9, and 5.1%. The above results suggested that the fermentation of *A. membranaceus* by *E. faecium* increased the abundance of *Aspergillus*, while the fermentation of *A. membranaceus* by *E. faecium* or *L. plantarum* reduced the abundance of *Penicillium* and *Cystofilobasidium*. Moreover, *L. plantarum*-fermentation of *A. membranaceus* significantly increased the abundance of unclassified genera.

### Species-Level Compositions of the Endophytic Fungal Communities

**Figure 2:**
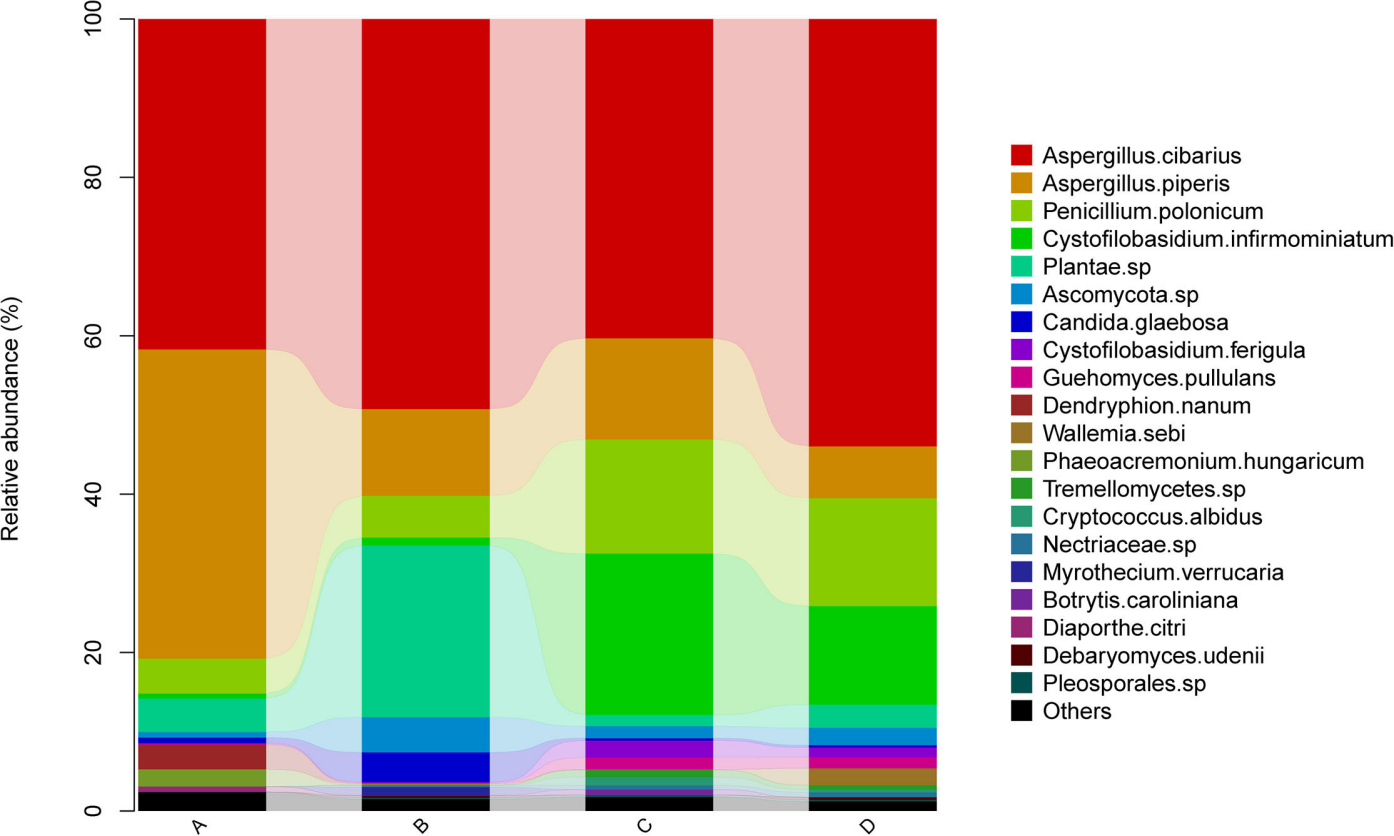
Species levels in samples. *membranaceus* samples under fermentation with *E. faecium* (A), *L. plantarum* (B), *E. faecium* + *L. plantarum* (C), and LBS (D) on day 6.

Endophytic fungal species in *A. membranaceus* were mainly *Aspergillus cibarius, Aspergillus piperis, Penicillium polonicum*, C*ystofilobasidium infirmominiatum*, and *Plantae* sp (Figure 2). The abundances of *Aspergillus cibarius* in the 4 groups were 41.7, 49.3, 40.3, and 54.0%. The abundances of *Aspergillus piperis* were 39.1, 11.0, 12.8, and 6.5%. The abundances of *Penicillium polonicum* were 4.4, 5.3, 14.4, and 13.6%. The abundances of C*ystofilobasidium infirmominiatum* were 0.6, 1.0, 20.4, and 12.5%. The abundances of *Plantae* were 4.2, 21.7, 1.4, and 2.9%, respectively. The above results show that the fermentation of *A. membranaceus* by *E. faecium* mainly increased the abundance of *Aspergillus piperis* and reduced that of *Penicillium polonicum* and *Cystofilobasidium infirmominiatum*. Meanwhile, fermentation of *A. membranaceus* by *L. plantarum* reduced the abundance of *Penicillium polonicum* and *Cystofilobasidium infirmominiatum* but increased that of *Plantae*.

### Cluster Analysis

**Figure 3:**
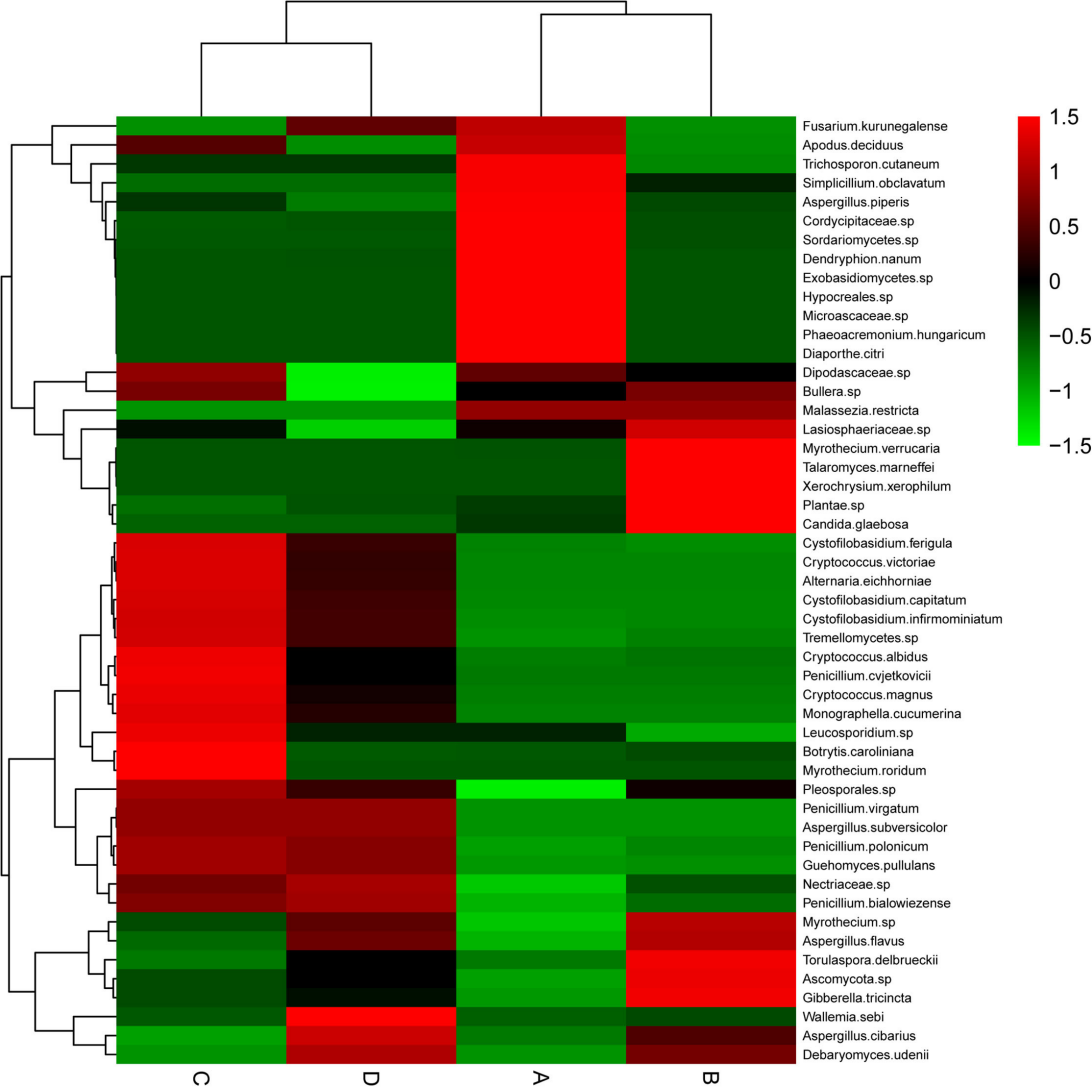
Heat map of endophytic fungal community composition with cluster analysis. *A. membranaceus* samples under fermentation with *E. faecium* (A), *L. plantarum* (B), *E. faecium* + *L. plantarum* (C), and LBS (D) on day 6. Green and red colors stand for low and high abundances, respectively.

This study clustered those 50 species with the highest abundances by R software. As revealed by the heat map (Figure 3), the highly abundant species of fungi in groups A, B, and C were much better than those in group D. Group A had a higher abundance of *Trichosporon cutaneum, Simplicillium obclavatum, Aspergillus.piperis, Cordycipitaceae sp., Sordariomycetes sp., Dendryphion nanum, Exobasidiomycetes sp., Hypocreales sp., Microascaceae sp., Phaeoacremonium hungaricum*, and *Diaporthe citri*. Group B had a higher abundance of *Myrothecium verrucaria, Talaromyces marneffei, Xerochrysium xerophilum, Plantae sp., Candida glaebosa, Torulaspora delbrueckii, Ascomycota sp*., and *Gibberella tricincta*. In group C, *Cryptococcus albidus, Penicillium cvjetkovicii, Cryptococcus magnus, Monographella cucumerina, Leucosporidium sp., Botrytis caroliniana*, and *Myrothecium roridum* were the most abundant species. However, only *Wallemia sebiand* and *Aspergillus cibarius* were particularly abundant species in group D. The above results demonstrated that the endophytic fungal abundance of *A. membranaceus* can be changed by fermentation, and the change was closely related to the bacteria used for fermentation.

## Discussion

Endophytic fungi are extensively distributed within healthy plant organs, which are the critical parts in the plant micro-ecosystems during the long-term evolution, significantly influencing plant metabolite production and affecting medicinal plants-derived crude medicine quantity and quality (7). At present, the cultivation method is primarily adopted for investigating endophytic fungal diversity, but this method is limited by the low authenticity of endophytic fungal community composition. With the rapid development of sequencing technology, SMRT sequencing can more accurately describe microbial diversity (17). Previously, 16S rRNA gene-SMRT sequencing was conducted for analyzing *A. membranaceus* diversity fermented by *L. plantarum* and *E. faecium*. As a result, it was appropriate to assess fermented *A. membranaceus* quality using the SMRT sequencing system (3). For exploring how *E. faecium* and *L. plantarum* fermentation affected the endophytic fungi of *A. membranaceus*, 18S rRNA gene-SMRT sequencing was used for analyzing *A. membranaceus* fermented endophytic fungal composition and diversity.

Based on the ACE, Chao, and Shannon indices, *A. membranaceus* fermented by *L. plantarum* possessed the highest fungal community diversity. Furthermore, *A. membranaceus* fermented by *L. plantarum* exhibited the highest number of OTUs by sequencing, suggesting that *L. plantarum* fermentation increased the abundance of endophytic fungal diversity of *A. membranaceus*. In general, the endophytic fungal diversity of *A. membranaceus* was relatively low, independent of fermentation. Composition analysis of the endophytic fungal community showed that the dominant fungal phyla of *A. membranaceus* were *Ascomycota* and *Basidiomycota*, and the abundance of *Ascomycota* was increased by *E. faecium* fermentation and decreased by *E. faecium and L. plantarum* fermentation. However, *E. faecium and L. plantarum* fermentation increased the abundance of *Basidiomycota*. *Aspergillus* was the genus with the greatest relative abundance among the endophytic fungi of *A. membranaceus*. In Asian cuisines, *Aspergilli* represent the critical microorganisms that can enhance the flavors, nutrients, and textures of fermented foods (18). Moreover, the use of *Aspergillus* fungus for fermenting the native *A. membranaceus* significantly elevated phenolic levels of *A. membranaceus*; besides, *A. membranaceus* after fermentation displayed significantly increased antioxidation capacity (19). According to our results, the fermented *E. faecium* had markedly increased *Aspergillus* abundance. Overall, the results showed that fermentation can change the proportions of endophytic fungi of *A. membranaceus*, which might change the metabolite contents of *A. membranaceus* processed by fermentation.

A recent study on the saponins of *Dipsacus asperoides* showed that endophytic fungi from taproots generated metabolites with bioactivity close to plant hosts, and their quantity showed positive relation to *Dipsacus* saponin content (20).

Some studies used high-throughput sequencing technology to analyze and identify the endophytic fungal community of *A. membranaceus*, explored the diversity information of endophytic fungi of *A. membranaceus*, solved the limitations of non-culturable and low-abundance fungi that could not be detected in traditional methods, and provided a reference for the use of endophytic fungi in *A. mongholicus* for pure fermentation or mixed fermentation to transform glycosides in *Astragalus*.

High-throughput sequencing in this study may provide a partial answer to previous research demonstrating that the active substances of *A. membranaceus*, such as *Astragalus* polysaccharides, flavonoids, saponins, and organic acids, are changed by *L. plantarum and E. faecium* fermentation (3). Recently, endophytic fungi originating from *Astragalus* species have been used for the biotransformation of *Astragalus* sapogenins (21). *A. chinensis*-derived endophytic fungi are verified to be bactericides and fungicides (22). It is exciting to speculate that *Astragalus* polysaccharides or flavonoids may be obtained from the endophytic fungi that colonize *A. membranaceus*. Our findings provide evidence for this possibility.

In summary, 18S rRNA gene-SMRT sequencing was used for analyzing endophytic fungal composition and diversity from fermented *A. membranaceus*. Our study presents the most comprehensive data for a more extensive follow-up study.

## Data Availability Statement

The original contributions presented in the study are included in the article/supplementary material, further inquiries can be directed to the corresponding author/s.

## Author Contributions

YG and XK shouldered the responsibility of studying conception and design. LZ and XL were in charge of experiment implementation. XS and HQ were responsible for statistical analysis and were in charge of manuscript writing. JZ and CB isolated and preserved microbial strains. All authors revised the study and approved the final version for submission.

## Funding

This work was supported by the Natural Science Foundation of Henan Province of China (202300410189), the Research and Innovation Team of Henan University of Animal Husbandry and Economy (2018KYTD13), and the Key Discipline of Preventive Veterinary Medicine of Henan University of Animal Husbandry and Economy (mxk2016102 and 2021HNUAHEDF004).

## Conflict of Interest

The authors declare that the research was conducted in the absence of any commercial or financial relationships that could be construed as a potential conflict of interest.

## Publisher's Note

All claims expressed in this article are solely those of the authors and do not necessarily represent those of their affiliated organizations, or those of the publisher, the editors and the reviewers. Any product that may be evaluated in this article, or claim that may be made by its manufacturer, is not guaranteed or endorsed by the publisher.

## Abbreviations

*A. membranaceus*: *Astragalus membranaceus*
*L. plantarum*: *Lactobacillus plantarum*
*E. faecium*: *Enterococcus faecium*
SMRT: single-molecule, real-time
CFU: colony forming units
OTUs: operational taxonomic units
PCA: Principal component analysis.
